# Spatial confluence of psychological and anatomical network constructs in the human brain revealed by a mass meta-analysis of fMRI activation

**DOI:** 10.1038/srep44259

**Published:** 2017-03-13

**Authors:** William Hedley Thompson, Peter Fransson

**Affiliations:** 1 Department of Clinical Neuroscience, Karolinska Institutet, Stockholm, Sweden

## Abstract

It is well-known that the brain’s activity is organized into networks but it is unclear how many networks exist. Additionally, there is also a risk of ambiguity since different names for the same network are frequently reported in the literature. In this study, we employed a mass meta-analysis of fMRI data associated with network constructs originating from both psychology and neuroscience. Based on the results from the meta-analysis, we derived a spatial similarity map between all construct terms, showing that the brain’s networks cluster hierarchically into several levels. The results presented are useful as a first step in developing a unified terminology for large-scale brain network and a platform for a queryable network atlas.

There is a general acceptance that cognitive processes are distributed across anatomically wide-spanning networks in the human brain^1^,^2^. Let us begin by posing a simple question: how many large scale networks does the human brain have? Although it is simple to formulate, it is a question that cannot be answered in a straightforward manner. Theoretically, the maximum number of brain networks could be one for every cognitive, psychological and behavioral phenotype. When considering the diversity of network assignments that can be found within the neuroscientific literature, one would perhaps be inclined to think that this theoretical maximum is valid. If we consider a certain psychological process of interest that is examined in a functional neuroimaging context (let’s call it “x”), it is very likely to result in a spatially distributed pattern of brain activity. The process “x” can range from referring to rather general psychological functions (e.g. a multi-sensory network^3^), to specific psychological functions (e.g. empathy network^4^), to specific behaviour networks (e.g. smooth pursuit eye movements network^5^) to even more abstract behavior (e.g. mindfulness meditation network^6^). Thus, there is a strong tendency in the literature to adopt network constructs to describe spatial distributed brain activation patterns obtained from functional neuroimaging studies. It is also noteworthy that the naming of candidate functional networks varies widely in the literature. Returning to the question above, is the theoretical maximum of networks reasonable to assume where the brain has developed a specialized network for every conceivable variation of cognitive processes? This possibility strikes us as unlikely.

If we instead assume that no one-to-one mapping between network and cognitive process is likely to exist, it is to be expected that different networks are identified and named differently depending on the specifics of the experimental context that was used to identify them. Thus, the present lack of consensus in how to name brain networks is understandable but troublesome. The naming of large scale networks may have at least two different origins. First, specific patterns of brain activity may be classified from a psychological point of view into what could be described as psychological network constructs (PNCs), e.g. “reward networks”^7^ and “episodic memory network”^8^. The second kind of network constructs originates in brain anatomy and/or function (brain-based network constructs, BNCs), for example “fronto-parietal network”^9^ and “default mode network”^10^. For these particular networks, the anatomical or functional (correlations of brain activity) structure are identified in absence of a specific psychological function. Once identified, BNCs may be attributed to one or several cognitive process.

Neither of these network naming strategies are unproblematic. PNC’s, albeit intuitive, suffer from being vaguely defined and their uniqueness is questionable, e.g. how similar/dissimilar is the “fear network” from the “affect network”? The consequence is overlapping, ambiguous or duplicate network names. On the other hand, BNCs also suffer from ambiguity since no unique atlas of networks exists, and there is no general agreement on how to systematically map psychological faculties (or PNCs) onto BNCs. Additionally, there are several instances when it becomes unclear whether a given brain network should be labeled a PNC or a BNC (e.g. the “visual network” appears in behavioral/psychological contexts of vision processes as well as in functional brain network contexts where it can be reliably detected using resting state fMRI). While both PNCs and BNCs have been proven useful to understand the complex relationship between mental processes and brain anatomy and function, the current state of affairs is problematic since different BNC and PNCs are embraced depending on the specifics of experimental context, which results in an adoption of different definitions and boundaries that reflect particular sub-fields of brain research.

To prevent naming ambiguity and promote consensus, are there any tools for researchers to identify which network their functional imaging results belong to, in any systematic way? While researchers frequently report brain coordinates and anatomical names, since researchers have several tools and atlases available for reporting anatomical regions (e.g. Brodmann or AAL areas^11^), no such tool exists for relating functional imaging results to large-scale networks. Instead researchers often fall back to the problematic inference practice of “if brain area A, then network B.” In order to promote reporting of results in terms of networks, tools are needed which allow for researchers to infer the underlying network(s). For such a tool to exist, steps have to be taken to move towards a unified terminology.

Returning again to our initial question, this study does not aim to answer how many networks the brain has. Rather, our intention was to investigate two aspects of the relationship between network naming and brain activity patterns that we believe are of central importance^1^. The spatial topography of PNCs and BNCs cluster in a hierarchical order based on their voxel activation patterns^2^, the same anatomical region can be involved in multiple networks. To address these issues we present a data-driven method to unify the naming process of brain networks and thereby promoting a platform for a naming consensus of brain networks.

Our aims are not without precedence. The exact relationship between the diversity of different mental processes and anatomical networks is indeed an ongoing area of research. Different approaches to the cognition-brain network anatomy correspondence problem has been proposed. The two main tools used in these explorations are BrainMap^12^ and Neurosynth^13^, which both are meta-analytical tools that contain functional imaging data harvested from several thousands of articles^14^,^15^. The results from these investigations are interesting. Using BrainMap, it has been shown that resting state networks overlapped with activations during various tasks^16^ which was extended and a hierarchical structure of meta-data associated with experiments was shown to correlate with the networks derived using independent component analysis^17^. Brain activity patterns have also been dubbed “functional fingerprints”, and these fingerprints, containing specific activation patterns, correspond to different cognitive functions^18^. More recently, a study identified modules in the brain by clustering voxels and meta-data, and then identified which voxels were present within multiple modules^19^. Using Neurosynth, researchers have also clustered terms into “topics” of brain activation and mental disorders^20^ and then used reverse inference to reveal associations to any given voxel^21^. Further, Neurosynth has also been used to assign unique anatomical areas to functions such as pain (ref. 22 for its controversy see refs 23 and 24). Our work partly overlaps with several of these previous studies. However, in contrast to the hierarchical structure outlined in ref. 17, we derived our results based on the similarity between voxel activation patterns instead of meta-data clustering, which enabled us to detect considerably more networks than previously reported. Importantly, using different methodology and clustering techniques, we were able to identify voxels that are active in multiple cognitive tasks as in ref. 19.

We used Neurosynth^13^, a meta-analysis tool that derives fMRI activation maps based on network constructs associated with brain activity reported in the literature (11.406 fMRI studies included as of December 1, 2015). An initial list of 3107 terms was filtered to include only terms with a least one hit in the PubMed database for the search phrase “<term> network” (see Methods). After filtering, 148 terms remained which represented plausible network constructs. These included PCNs such as “self” and “empathy” as well as BCNs such as “occipito-temporal” and “temporal-parietal”. For each term pairing, we performed a reverse inference meta-analysis (FDR corrected at p < 0.01) identifying fMRI spatial activation profiles stored in Neurosynth. Each pair-wise comparison between search queries *i* and *j* was conditional, ensuring that no article contributed to both activation maps. Subsequently, the spatial overlap of brain activity associated for each pair of terms was calculated. The degree of spatial overlap in the brain between terms *i* and *j* was quantified as the percentage of voxels *j* shared with *i*. This was done for each term pairing and resulted in a 148 × 148 spatial similarity matrix (*O*). Edges with a non-significant overlap were set to zero (p < 0.05 see Methods and Supplementary Figure S1). The resulting connectivity matrix is shown in Fig. 1a. Using this connectivity matrix we aimed to explore the similarity of network constructs as reported in in the literature and derive data-driven candidate “template” networks.

## Results

**Figure 1:**
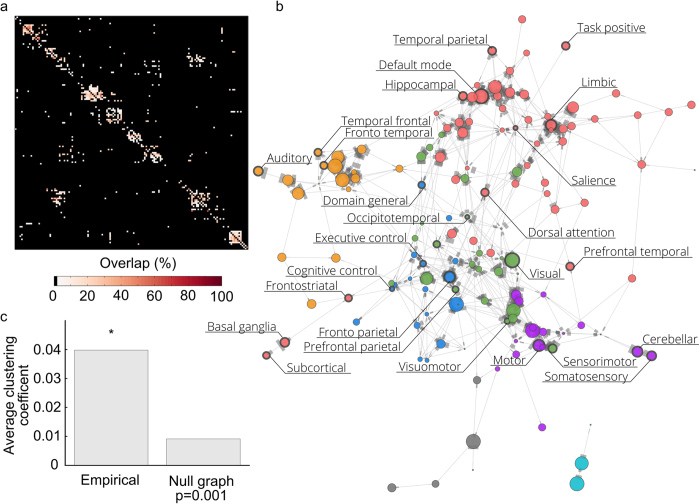
Clustering of spatial similarity between network constructs. (**a**) Similarity matrix *O*_*ij*_ displaying the degree of similarity between network terms after pruning. (**b**) An edge-weighted spring embedded layout of (**a**), where each circle denotes a network term. The size of the circle is determined by the weighting terms *w*, which is the difference between how much network term *i* overlapped with other terms. Candidate BNCs are marked with a thicker black border and have their names shown. An interactive version of Figure 1b can be downloaded at:  https://github.com/wiheto/MMA_of_brain_networks (**c**). Average clustering coefficient of the similarity matrix (marked as ‘empirical’) versus the average clustering coefficient of the permuted null model at p = 0.001. The asterisk marks that the similarity matrix had a significant average degree of clustering compared to the null graph.

The spatial similarity matrix, *O* yielded by the mass meta-analysis of fMRI activation patterns, is shown in Fig. 1. From Fig. 1a, we observe a certain degree of structure in the similarity matrix, indicating clustering of similarity between network constructs. The presence of clustering was statistically assessed by testing the weighted local clustering coefficient, averaged over all nodes in *O* (C = 0.0398) and comparing this against a null graph (Fig. 1c, significant at p < 0.001).

Given the significant degree of clustering in *O*, the next step was to determine the structure of the clustering by identifying communities. To this end, the hierarchical version of the InfoMap algorithm was used^25^. The hierarchical version of InfoMap outperformed the non-hierarchical version (62.9% compression, with 7.4% greater compression than the non-hierarchical version of InfoMap). Thus, the clustering in *O* adheres more to a hierarchical clustering.

With the hierarchical InfoMap algorithm each node in *O* can be seen as a leaf (i.e. terminal node) in a tree structure. At the first level, nodes are clustered together. Then, in a recursive fashion, these clusters of nodes are further clustered together into larger clusters. This results in layers of clusters spanning multiple levels, forming a hierarchy. Each cluster contains a set of nodes from *O*. Nodes in *O* can only belong to one cluster per hierarchical level. Here, the InfoMap algorithm yielded up to three hierarchical levels. For ease of reference, we refer to individual clusters of spatial networks using the following nomenclature: Cx to refer to the x:th cluster at the 1st level, Cx.y to refer to the y:th cluster at the 2nd level, and Cx.y.z to refer to the z:th cluster at the 3rd level (if present). Note that levels of clustering are arranged according to spatial size of the clusters, meaning that larger clusters are found at the 1st level whereas increasingly smaller clusters are found at the 2nd and 3rd levels. Clusters on the 1st level will then contain all the clusters in the levels below and be considerably larger and more general than the smaller clusters.

As a first step to illustrate the degree of clustering among spatial BNC and PNC networks, we have in Fig. 1b included a spring-embedded plot where each network construct is represented as a node and its color symbolizes cluster membership at the first level^26^. From Fig. 1b it can be observed that clusters of network constructs include both PNC and BNC terms. Moreover, a substantial degree of spatial overlap can be seen, for example “attention” overlaps with “fronto-parietal” and “imitation” overlay with “sensorimotor”. The BNC “default mode” overlaps with PNCs such as “self” and “autobiographical memory”. An interactive version of Fig. 1b can be downloaded at: https://github.com/wiheto/MMA_of_brain_networks. While many similarities between these terms was to be expected, our results provide a systematic overview of the spatial relationship between PNCs and BNCs.

**Figure 2:**
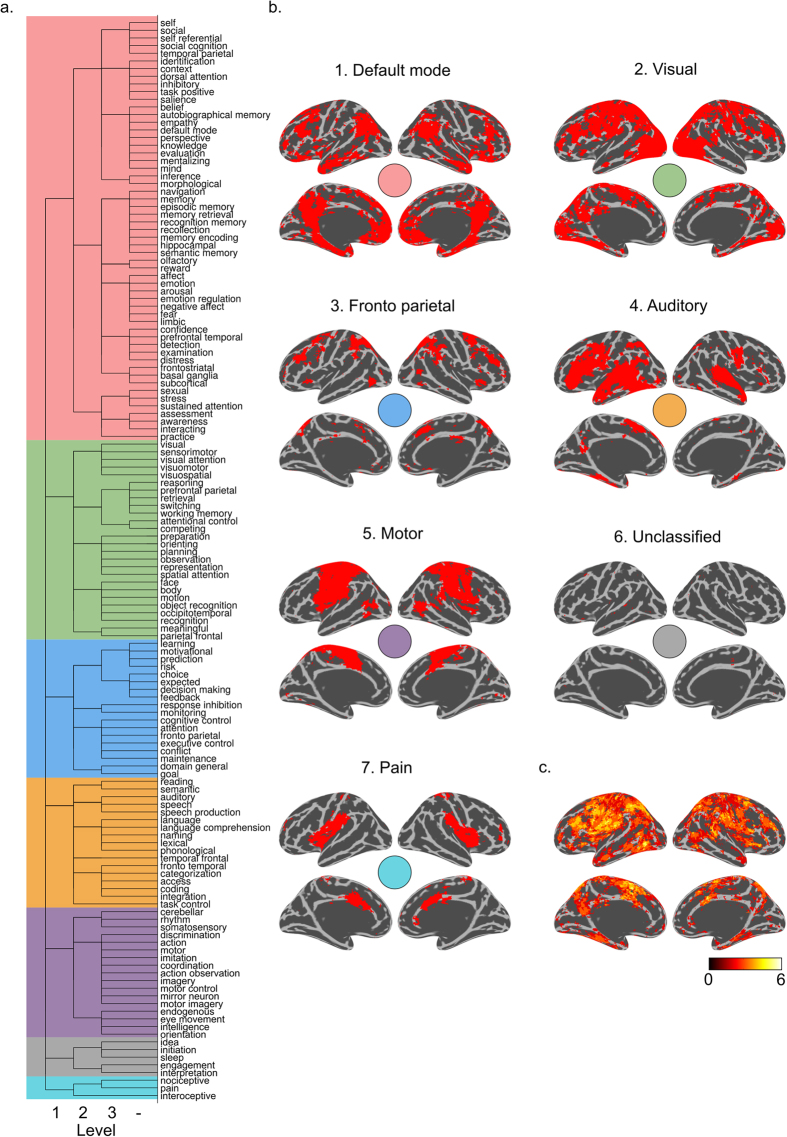
Hierarchical clustering of the spatial similarity of network constructs in the brain. (**a**) Tree plot showing three levels of branching. Background color depicts the first level of clustering. (**b**) Masks of all voxels included in any of the terms within the first level of clustering. The name given for each first level cluster in Fig. 2b is the BNC with the largest weighting factor *w* of the first level clusters, with the exception of C7 which did not included a BNC and instead shows the largest *w* of any of the terms. C6 was ambiguous in both spatial patterns and collection of network terms and was left unclassified. (**c**) Spatial overlap of all seven first level clusters projected onto the cortex surface. Colors denote how many of the first level cluster masks that are present at any particular voxel. All colors indicate at least 2 clusters are present.

Next, we aimed to analyze the degree of cluster hierarchy and the results are shown in Fig. 2a in the form of a tree diagram. We observe that some clusters (e.g. a memory, an emotion cluster a default mode network cluster) are all contained within C1, and each branch off into separate clusters at the 2nd level. To get an overall picture of the spatial confluence at the 1st level, we created binary masks for each cluster, where a voxel was marked as 1 if it was found in any of the network terms within its own cluster, and 0 otherwise. The binary network masks are shown in Fig. 2b. For 6 out of the 7 network masks shown in Fig. 2b, we note distributed patterns of activity which show a spatial resemblance to brain networks that previously has been reported in the literature. To examine the spatial structure of networks and investigate the degree of spatial overlap for all clusters of networks, we superimposed all masks and rendered them onto a single brain surface, illustrating which brain areas are involved in at least two different clusters of networks at the first level (see Fig. 2c). The spatial confluence of the terms (network constructs) shown in Fig. 2c suggests that brain regions distributed across large portions of the cortex are involved in multiple large-scale networks. For instance, voxels in the medio-posterior parietal cortex, insular cortex, supplementary motor cortex, temporal and dorso-lateral prefrontal cortex (DLPFC) are participating in multiple clusters of networks. This implies that these brain regions have a large degree of flexibility in terms of involvement in networks that are members of different psychological network constructs.

**Figure 3:**
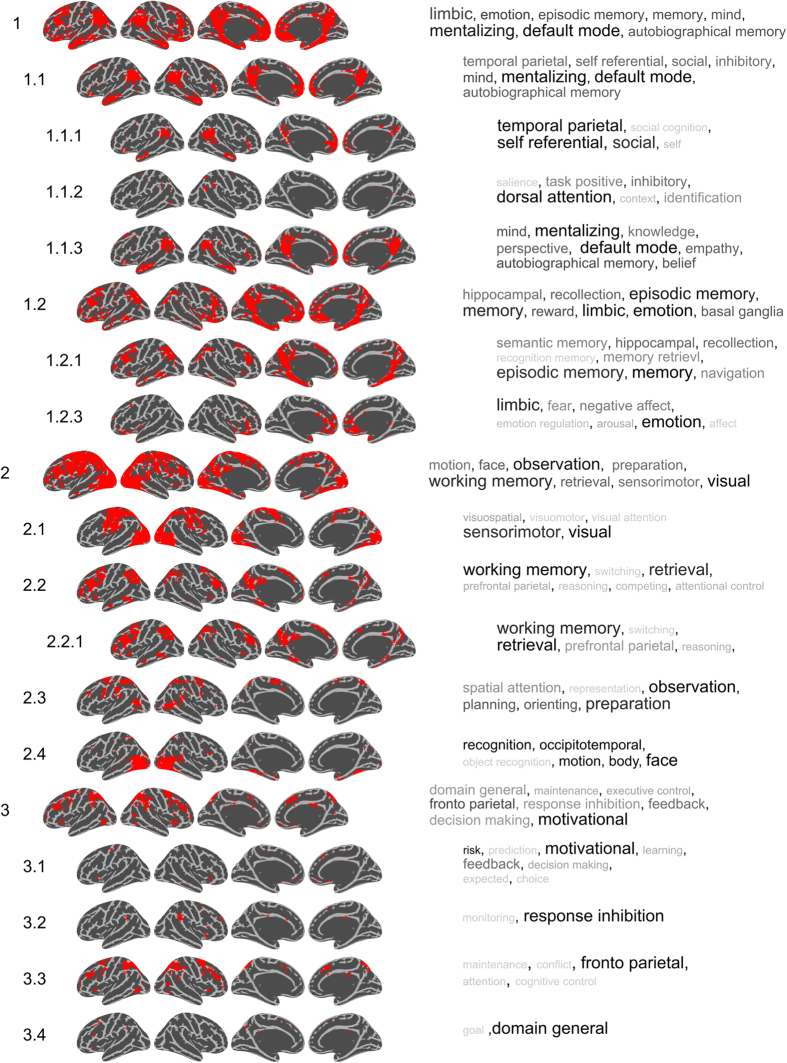
Binary masks of the hierarchical clustering of network constructs (see also Fig. 4). Masks are only shown if ref. 1 they encapsulate more than one network term^2^, included network terms and a spatial map that was judged to be reasonable. Due to the subjective nature of ref. 2, the excluded maps are shown in Supplementary Figure 2. The naming of the clusters is given to the left and reflects the cluster assignment where a period indicates a new cluster level (also illustrated by the indentation in the Figure). To the right of each spatial mask is a pseudo-word cloud included of the terms that are contained within each cluster and where size and color intensity reveal which terms relative to the other terms overlap with most other terms in the cluster (weighted by *w*, see also Methods section). The calculation of the weighting factor, *w* and the scaling of *w* was done independently for each cluster at each level.

**Figure 4:**
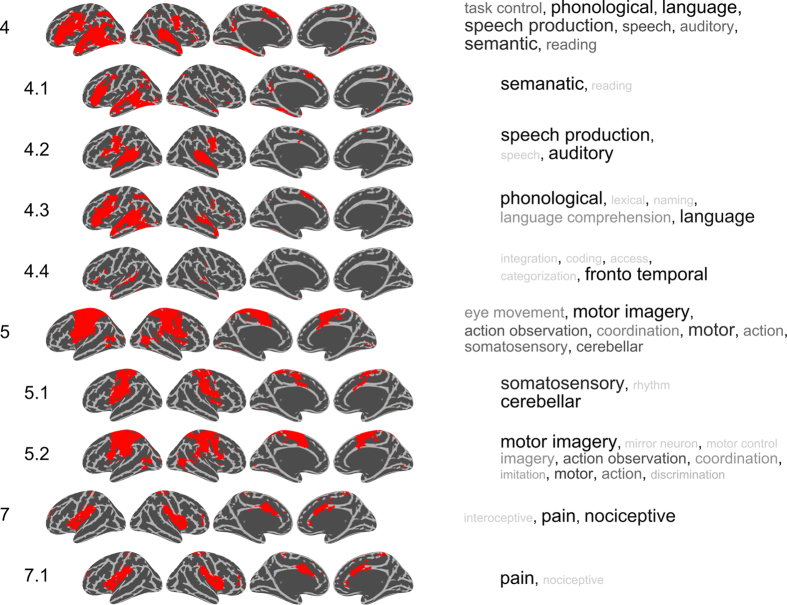
Same as Fig. 3 for the remaining clusters with branches starting at C4, C5 and C7.

Our next step was to analyze the 2nd and 3rd levels in the hierarchical clustering of spatial networks. Figures 3 and 4 show clusters that were interpreted to have meaningful networks on the cortex with a non-ambiguous collection of network terms in each cluster. From the results presented in Figs 3 and 4 we note that more general-purpose networks are found at the higher levels of clustering. For example, cluster C4 contains language related terms, but at the second level, clusters C4.1 and C4.2 split up into a semantic and reading network that are separated from the auditory, speech and speech production networks. Cluster C1.1.1 includes a “temporal parietal” BNC together with a “self” and a “social cognition” PNC, while C1.1.3 splits into a cluster containing the “default mode” BNC as well as PNCs that encompasses “mentalizing” and “autobiographical memory”. The results from the hierarchical clustering shown in Figs 3 and 4 allowed us to differentiate which networks are spatially similar, at both a coarse scale (level 1) as well as detailing more nuanced differences (at levels 2 and 3). A list of rejected clusters and their spatial distributions are given in Supplementary Figure 2.

Finally, the results shown in Figs 2–4 suggests that multiple networks are present at different levels in a hierarchy. Provided the demonstrated links between spatial network activation patterns and PNC/BNC constructs that are associated with each of them, it is obviously tempting to given each cluster a unique name. However, giving names to each of the spatial clusters shown here may be rather subjective. In this study, we provide a more data-driven naming suggestions by calculating a naming weight for each network term, *w* (see eq. 2 in the Methods section). The weight of each network term is shown to the right of Figs 3 and 4, where the color and size scale with *w* (limited to max 8 in the Figures for presentation purposes). The weights reveal which network terms that have spatial masks associated with them that encompass masks from the other terms within their cluster more than they are included in other network terms.

The network masks shown in Figs 3 and 4 are surface renderings of the cortex and therefore fail to display the differences located outside the cortex. For example, although Fig. 3 shows that clusters C1.2.1 (memory) and C1.2.3 (emotion) cover different areas of the cortex, considerable differences in the hippocampus and amygdala are not visible in Figs 3 and 4. Another example is cluster C3.1 (motivational, risk, feedback, learning) which has considerable degree of activation in the basal ganglia which is not apparent from Fig. 3. To facilitate a full picture of all involved brain aras, Figs 3 and 4 can be downloaded at https://github.com/wiheto/MMA_of_brain_networks as nifti files. The downloadable versions of the cluster network masks are included in a “_selected” version which are equivalent to the spatial maps shown in Figs 3 and 4. Furthermore, we have also included masks in an “_all” variant, which includes rejected clusters and are provided as a means to limit the amount of subjective decisions included in our selection of network terms and provide researchers with full set of tools to utilize and help assign their results in relation to the network patterns described here.

## Discussion

To recapitulate, we had three aims in the present study. 1. To illustrate that there is a spatial hierarchy in terms of spatial brain network that originate in psychological as well as anatomical constructs. 2. To show that many brain areas are involved in multiple networks. 3. To provide a first step towards obtaining a useful tool that can be employed to systematically report brain activity in a framework of an atlas of brain networks.

Regarding the first aim, we have shown that there is a considerable degree of spatial overlap between different psychological network constructs currently used in the neuroimaging literature and that the spatial architecture of networks is organized in a hierarchical manner. Our findings suggest that there is need for a data-driven and unifying naming scheme for brain networks, or perhaps even better, a comprehensive atlas of large-scale brain networks. Moreover, our results suggest that markedly different cognitive processes may be mapped onto similar large-scale networks. For example, this many-to-one relationship can be observed for terms such as the “social” and “self” networks, which are both within the same 2nd level cluster (Fig. 3). It might then be pertinent to ask whether it makes sense to claim that there is a unique “self network” or a unique “social network”? The same argument can be made for the network constructs “executive control”, “cognitive control”, “attention”, and “fronto parietal”. This situation calls into question an implicit assumption that often is made, namely that a one-to-one correspondence between brain networks and psychological network constructs exist. While the results presented here might only provide a first step towards a more unified way to think about the relationship between spatial brain network and cognitive function, we believe that we in the future also need to take into account the temporal dynamics of within- and between network connectivity to have a full understanding of the relationship between the brain’s repertoire of networks and cognitive functions. By, albeit subjectively, dividing network construct terms into BCNs and PCNs we have illustrated that most clusters of networks have at least one possible BCN attached to several different PCNs.

Our second aim was to show that single anatomical brain regions may contribute to multiple networks. We do not believe that this claim is controversial. Our results show that many brain regions are involved in multiple networks that span a wide array of psychological network constructs (see Fig. 2c). While this is already known and it has previously been shown convincingly in ref. 19, we hope that our results further deter from reasoning along the lines of “activation in DLPFC implies attentional network”. Further, this speaks against the notion that certain brain regions can be reduced to singular psychological constructs, as recently advocated in ref. 22. We argue that the entire spatial context surrounding a given set of brain activations is required to fully infer function from neuroimaging. To this end, we hope that the present results may be used to identify and communicate which brain networks that are involved in any set of brain activation maps obtained in fMRI studies. We see this work as a first step in developing tools for researchers for the purpose of identifying brain networks and a unification of the naming terminology used. The spatial network maps presented here are available online (www.github.com/wiheto/ANL).

Regarding the third aim, while we have presented a data-driven mechanism for classifying brain activation data in a network perspective, we are not advocating the usage that the top term of every cluster should be adopted. Hence, we do not think that the term “motivational network” should replace the “fonto-parietal network” term. Rather, we suggest that our results and the terminology presented herein might be useful to identify which networks that are involved in any given brain activation data set. For example, if a fMRI experiment yields a brain activation pattern that shows a strong spatial resemblance to the network cluster C1.1.1 (Fig. 3), we hope that the results presented here provides a good argument to describe the corresponding the results in terms of the BNC “temporal parietal network” rather than the PNC “social cognition network”. Thus, we would like to recommend that BNCs instead of PNCs are used whenever possible, as BNCs are not exclusive to PNCs, but PNCs may appear exclusive to other PNCs (e.g. “self referential network” seems to exclude the PNC “social cognition network”).

There are several drawbacks to the Neurosynth meta-analysis methodology^15^ that needs to be taken into account. Foremost, it should be mentioned that Neurosynth’s identification of target search terms in the literature is context-insensitive, which may have influenced our analysis of the degree of spatial overlap between network constructs. Perhaps less of a concern is the fact that two network constructs (“ventral attention network” and “cingulo-opercular network”) that are rather frequently used in research that targets the so called resting-state networks were missing from the Neurosynth database. This minor omission could potentially be remedied by formulating more complex search terms such as “ventral AND attention” and “cingulate AND opercular”, but at the present time we do not think it was justified to create custom search terms for just these two network constructs. Furthermore, despite the conditional strategy used here, we cannot rule out the possibility that we may still include instances in our meta-analysis for which the search terms *i* and *j* are present in the same studies when compared with another term *k*, which may bring terms *i* and *j* closer to each other in the similarity matrix. Not withholding the inherent methodological limitations of Neurosynth, our results on constructs of brain networks from the neuroimaging literature shows that a considerable degree of spatial clustering as well as a spatial overlap for different network construct is present in the brain (Fig. 2c).

In conclusion, based on data collected from a mass meta-analysis of previously published fMRI activation maps we derived a hierarchical clustering technique to construct a spatial similarity map of psychologically and anatomically rooted constructs of brain networks. Subsequently, we showed that the spatial similarity map could be divided into spatially overlapping clusters in the brain that adhered to a hierarchical ordering. Our results suggest that most spatial clusters of networks have at least one possible BCN attached to several different PCNs, which suggests a link between anatomically derived networks and networks that are grounded in psychology. Importantly, we present a purely data-driven approach to classify brain activations into networks, a step we believe will become increasingly important as the functional neuroimaging field of research is steadily progressing from a perspective dominated by localizing brain activation patterns into a a broader view of brain function that is based on the assumption of a dynamic interplay between different networks at different temporal and spatial scales. Finally, in the near future we aim to extend this work and to provide a publicly available tool that automatically, for any input brain activation nifti image, assign cluster memberships and network terms that are most closely associated with the input image, aiming to create an automated network labeling atlas that would help researchers to report their findings that is substantiated in the division of the brain into large-scale networks.

## Methods

### Query of the pubmed and neurosynth databases

Our meta-analysis of the neuroscientific literature used Neurosynth (www.neurosynth.org^13^). Our aim was to perform a meta-analysis on search terms that researchers had attributed to networks. We followed the following procedure. (1) All search terms available in the Neurosynth database (3107 unique terms as of December 1, 2015) were extracted. (2) For each of the terms, we queried the PubMed database using the text string “<term> network” and terms that generated at least one hit were kept. (3) The search results provided in step 2 were subsequently filtered using heuristics described below. (4) All network constructs were classed as PNC, BNC or both.

By querying PubMed for “<term> network” we limited ourselves to terms where researchers had, in at least one instance, reported that term in association with a network. This query resulted in 1027 unique terms that gave at least one hit in PubMed (also performed on December 1, 2015). However, many of the terms in both categories were rather vague or too general for the purpose of the present meta-analysis, or related to research fields outside neuroscience and were therefore not included in the analysis (e.g. “healthy”, “ventral” and “diagnostic”). When terms were perceived to express similar concepts, priority was given to terms that expressed a higher degree of specificity (e.g. “language comprehension” was chosen over “comprehension”). Anatomical terms that were deemed to not fulfill the criteria of what could be expected to constitute an anatomical network (e.g. “amygdala”) were also excluded while “basal ganglia”, “hippocampal” were included as they are often discussed in terms of networks. Additionally, terms related to brain pathology were excluded from the list. Moreover, anatomical network terms that encompassed larger sheaths of the cortex come in many flavors, for example, “fronto-parietal”, “frontoparietal” and “frontal parietal”. In such cases, all terms were included as one search term. Exceptions were made when the order of anatomical structures were reversed (e.g. “fronto temporal” vs. “temporal frontal”). Our reasoning for this was that such a naming convention may reflect the fact that different research subfields use these terms slightly differently and may therefore reflect different underlying cognitive process. After taking these considerations into account, 148 terms associated with the word “network” remained and were further analyzed using conditional meta-analyses based on fMRI activation maps acquired from Neurosynth. All 148 network terms are listed in Supplementary Table 1, which also includes a list of “additional terms” when more than one search term was included.

Broadly speaking, the extracted associations to the term “network” were assigned to belong to either one of two categories. The first category contained instances where the term “network” were used in the context of psychological network constructs (PNCs) such as “reward network”, “visual attention network” or “empathy network”. The second category included occurrences that were related to brain-based network constructs (BNCs), e.g. “fronto-parietal network”, “occipito-temporal” and “fronto-temporal”. The heuristic used for classification as a BNC was that it is a network which names an anatomic structure (e.g. “fronto-parietal) or frequently reported as being a resting state network in functional connectivity (e.g. default mode). Naturally, some network terms are harder than others to unanimously classify into being either a BNC or a PNC, respectively. For example, it could be argued that the term “visual network” could have connotations for both categories. Hence, for a minor part of our search terms, we attributed them to belong to both categories (see Supplementary Table 1 for a full list of classifications of all terms). Hence, our classification scheme should be considered as being an approximation, but we believe that the division of network constructs into these two categories is helpful for the interpretation of the results.

### Conditional meta-analyses of network search terms in the Neurosynth fMRI database

Without loss of generalization, let us consider the case of comparing just two networks. Regardless of their respective ontology (i.e. rooted in either psychology or anatomy), we wanted to estimate the similarity between them in the context of fMRI activation maps derived from a meta-analysis. Obviously, we wished to avoid the situation where studies included in the Neurosynth database contributed to both network search terms, as this will inflate the probability of overlapping activation maps. Therefore, we performed conditional meta analyses in Neurosynth by assuring that the same study never contributed for both search terms when computing the resulting fMRI activation maps. This was done by placing additional constraints when querying the Neurosynth database. Hence, for each comparison between network-terms *i* and *j*, an additional constraint was placed in the search string so that the other network was not included. Thus, the fMRI activity map for network term *i* was created by supplementing the search string so that it read “*i* and not-*j*”, and conversely, the fMRI activation map for network term *j* was based on the search string “*j* and not-*i*”.

The conditional search queries of the Neurosynth database ensured that the same studies never were used in both search terms when comparing the degree of similarity between fMRI activation maps. In cases where one of the network search terms contained the entire second network term, the conjunction and negation of the encompassing phrase was dropped. For example, the combination of the search terms “working memory” and “memory”, would result in the query “working memory and not-memory”. Obviously, this would produce in a nil result, since there is no study that include the phrase “working memory” which does not contain the phrase “memory”. In such instances, we used the longer term by itself. In the exemplified case, the two search terms would be: “working memory” and “memory and not-working memory”. This results in that neither search contained the same articles in their comparison. Consequently, conditional search queries in the Neurosynth database were performed for all pair-wise combinations of the 148 selected search terms.

The significant fMRI brain activity maps extracted from of the Neurosynth database were transformed into binary masks (reverse inference, voxels that passed the statistical significance threshold of p < 0.01, FDR corrected, as in Neurosynth^13^) were set to 1, all other voxels were set to 0). Deactivations were ignored.

### Assessing the spatial overlap of brain activity for network search terms

The spatial overlap of the binary fMRI map of network search term *j* with respect to term *i*, denoted 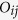 was calculated by:


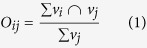


where *v*_*i*_ and *v*_*j*_ are the ensemble of voxels for which the binary masks equal 1. This measure gives the percentage of *j* 's voxels that overlap with *i*. This procedure was repeated for all search term comparisons entailing that *O* had the dimensions 148 × 148. If a spatial overlap between two network search terms failed to reach a statistically significant spatial overlap (see below), the corresponding entry in the similarity matrix was set to zero.

Of note, the measure stated in equation 1 provides a directional estimate of spatial overlap between networks with regard to differences in their individual sizes. To make this clear, consider the following scenario where term *j* consists of 5 voxels and term *i* consists of 100 voxels. If 4 voxels overlap, it makes more sense to let *O*_*ij*_ = 0.8 and *O*_*ji*_ = 0.04 rather than using an unidirectional measure (e.g Sørensen-Dice coefficient which would equal 0.076 for both connections). Thus, the directional measure allows us to see whether term *j* appears to be a part of *i* or vice versa.

### Testing for significant differences in similarity between network activation patterns

Since some pair-wise comparisons had very little overlap (e.g. 0.003%), we wished to prune the similarity matrix *O* so that it contained only relevant spatial similarities before testing for significant degrees of clustering and spatial overlap. To do the pruning, we performed a Monte Carlo non-parametric statistical test where our null hypothesis was framed as follows: “there exists no spatial similarity between the voxel-based fMRI activation maps that are associated with the network search terms *i* and *j*, respectively”. A schematic illustration of the permutation test is presented in Supplementary Figure S1. For the statistical test, we created null distributions in which the fMRI activation maps associated with terms *i* and *j* were randomly located across the brain. This null distribution allowed us perform statistical significance tests on *O*_*ij*_. For each pair of network terms, we started with two 3-dimensional binary fMRI activity maps. Each 3-dimensional map was transformed into a 1-dimensional vector where the cluster size and their within-map spatial adjacencies were preserved (see Supplementary Figure S1B). The length of the 1D vectors was 193,622 voxels, which equals the number of unique voxels inside the brain that showed fMRI activation patterns amongst any of the 148 network search terms defined. 1000 permutations were then made to create the null-distribution of spatial similarity. For each permutation, the clusters in each of the two vectors were shuffled to random locations within their respective vectors, maintaining the size of each cluster, and then the spatial similarity between the two vectors was calculated. *O*_*ij*_ was then compared to the null distribution and edges (i.e. degree of spatial overlap) were kept when the null hypothesis could be rejected at p < 0.05. This test is uncorrected for comparisons across multiple terms, which in turn may lead to a too small degree of pruning of *O*_*ij*_ being carried out. It should be noted that our main statistical inference (see below) is tested on clustering of *O* where spurious overlapping edges would be in favour of the null hypothesis.

An additional concern is that we are assuming a uniform probability for each voxel to be activated in our statistical test despite the fact that presumably some voxels have a greater likelihood of being activated than others in the fMRI literature. Thus, it cannot be ruled out that we may be allowing too many edges to remain as the chance of overlap may be less if a non-uniform probability of voxel activation was assumed in the permutation test. This leads to the possibility of too few edges are being pruned. But, again, as our main statistic inference is on the clustering coefficient, if additional spurious edges are included they will have a detrimental effect and bias the results towards there being less clustering in *O*.

### Graph theoretical measures of the degree of spatial similarity between network terms

Next, we were interested in whether the spatial similarity matrix encompassing all 148 network search terms showed a significant degree of clustering. The weighted directed clustering coefficient^27^ was calculated using the brain connectivity toolbox^28^ in Matlab 2014a (Mathworks). The local clustering coefficient for a node (here: network term) *i* quantifies the fraction of the nodes connected to *i* that are also connected to each other. A high value for the local clustering coefficient indicates greater clustering. Thus, a higher than chance degree of clustering between terms would suggest that some network constructs are more closely related to each other spatially in the brain than what would be expected by chance alone. To test this hypothesis, we set the null-hypothesis to “there is no significant clustering of the network terms in the similarity matrix *O*”, which was tested using a distribution of 1000 null models where the out-strength of nodes was preserved^29^. The null model was calculated 5 times, where each edge was rewired 1, 5, 10, 20, and 50 times, achieving the same results. A rewiring of 20 was used for the results shown Fig. 1c.

Once we had identified significant degree of clustering, we aimed to isolate and display these clusters. There are different methods available to achieve this aim. We used the Hierarchical InfoMap algorithm^25^, an extension of the InfoMap algorithm^30^. The original InfoMap clustering algorithm has been used in numerous studies in the neuroimaging literature (e.g. refs 31, 32 and 19). In essence, InfoMap aims to create efficient random walks through the network. The efficiency of the clustering is determined by calculating the number of bits needed for a random walk. To calculate the number of bits of a random walk, each node is assigned a unique name coded in a binary representation (Huffman coding^33^). These binary names are recorded when the algorithm randomly create walks through the network. Shorter binary names (e.g. 10) will require fewer bits to be stored when traveling through the network than longer names (e.g. 10100). The InfoMap clustering method allows for the reuse of node names by creating modules, and the same short name can be reused in different modules. Modules are also given a binary names and these are recorded when traveling between modules. This step entails that one always knows which module one is in during a random walk (so that no information is lost) and it also reduces the chance of too many modules being found, as the additional spurious between-module information would increase the number of bits needed. The reuse of shorter names and fewer between-module steps in the walk will reduce the number of bits recorded when walking through the network, with no information lost. A useful analogy that Rosvall & Bergstrom give is that we can reuse street names (e.g. “Vasagatan”) in multiple cities (Gothenburg and Stockholm). People use only the shorter street name without creating confusion instead of their longer names (“Vasagatan in Stockholm”). By creating names for clusters (cities) and names for each of the nodes (streets), we can travel between nodes and see how much information is saved by creating the city clusters, allowing ourselves to reuse the names. Thus, the aim of the InfoMap algorithm is to maximize compression of the random walks by partitioning the network into clusters. We used the hierarchical version of the InfoMap algorithm which is a generalization where the clusters, which are also assigned to binary names, can themselves cluster and cluster names can be reused (e.g. Gothenburg exists in both Sweden and America). For more information of the InfoMap algorithm see Rosvall & Bergstrom’s 2008 and 2011 papers. The algorithm was run 10,000 times to account for initiation differences. For clarity and consistency, instead of Rosvall and Bergstrom’s terminology of modules, we in this study use the term clusters.

## Weighting of network names

To generate weighting factors of each term’s membership for candidate name of clusters, we utilized the fact that *O* was directional. We derived a cluster centrality measure based on the difference of its input strength and output strength (*w*) which was computed by:


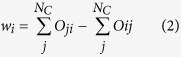


where 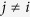. *N*_*c*_ denotes the set of nodes in a cluster and *i* and *j* are indexes of nodes. This weight is calculated for all nodes within a cluster. The weightings of a cluster were scaled between 0 and 1 relative only to the other nodes in the cluster. When a term is used in multiple levels of the cluster hierarchy, its value may differ as *w* is calculated with respect to the nodes in the cluster.

## Additional Information

**How to cite this article:** Hedley Thompson, W. H. and Fransson, P. Spatial confluence of psychological and anatomical network constructs in the human brain revealed by a mass meta-analysis of fMRI activation. *Sci. Rep.* **7**, 44259; doi: 10.1038/srep44259 (2017).

**Publisher's note:** Springer Nature remains neutral with regard to jurisdictional claims in published maps and institutional affiliations.

## Supplementary Material

Supplementary Information
