# Supplementary material for: Spatial confluence of psychological and anatomical network constructs in the human brain revealed by a mass meta-analysis of fMRI activation
Source: Sci Rep. 2017 Mar 13;7:44259. doi: 10.1038/srep44259 (PMC5347156; doi:10.1038/srep44259)
Supplement: Supplementary Information [file srep44259-s1.pdf]

Supplementary material

Spatial confluence of psychological and anatomical network constructs in the human brain revealed by a mass meta-analysis of fMRI activation.

William Hedley Thompson & Peter Fransson

Supplementary Figure S1. A schematic example that serves to illustrate how the statistical testing was conducted for each entry in the spatial similarity matrix (O_{ij}). Panel A of the Figure shows a schematic example of two possible binary fMRI brain activation patterns for search terms i and j, with 50% overlap (for simplicity and ease of visual illustration, the schematic brain activity maps in panel A are presented in a 2D space, but in reality they are obviously defined in a 3D space). For each permutation, the activation patterns shown in panel A were transformed into 1-dimensional vectors where the locations of active clusters of brain activity were randomly set (panel B). Note that the spatial adjacencies of the neighboring voxels inside clusters were retained. The permutation procedure for each combination of search terms i and j was repeated 1000 times to create a non-parametric distribution which could be used to test against a null hypothesis of no spatial overlap between search terms i and j.

Supplementary Figure S2: Excluded binary masks for clusters that were deemed as ambiguous (due to either their spatial pattern on the cortex or the collection of terms). Compliments Figure 3 and Figure 4.

Supplementary Table 1: Information regarding all search terms (nodes) used, including terms, alternative 18 terms used when searching the Neurosynth meta-data base, clustering coefficient for each nodes, cluster assignment, and weighting factor w at the first level cluster, scaled between 0 and 1.

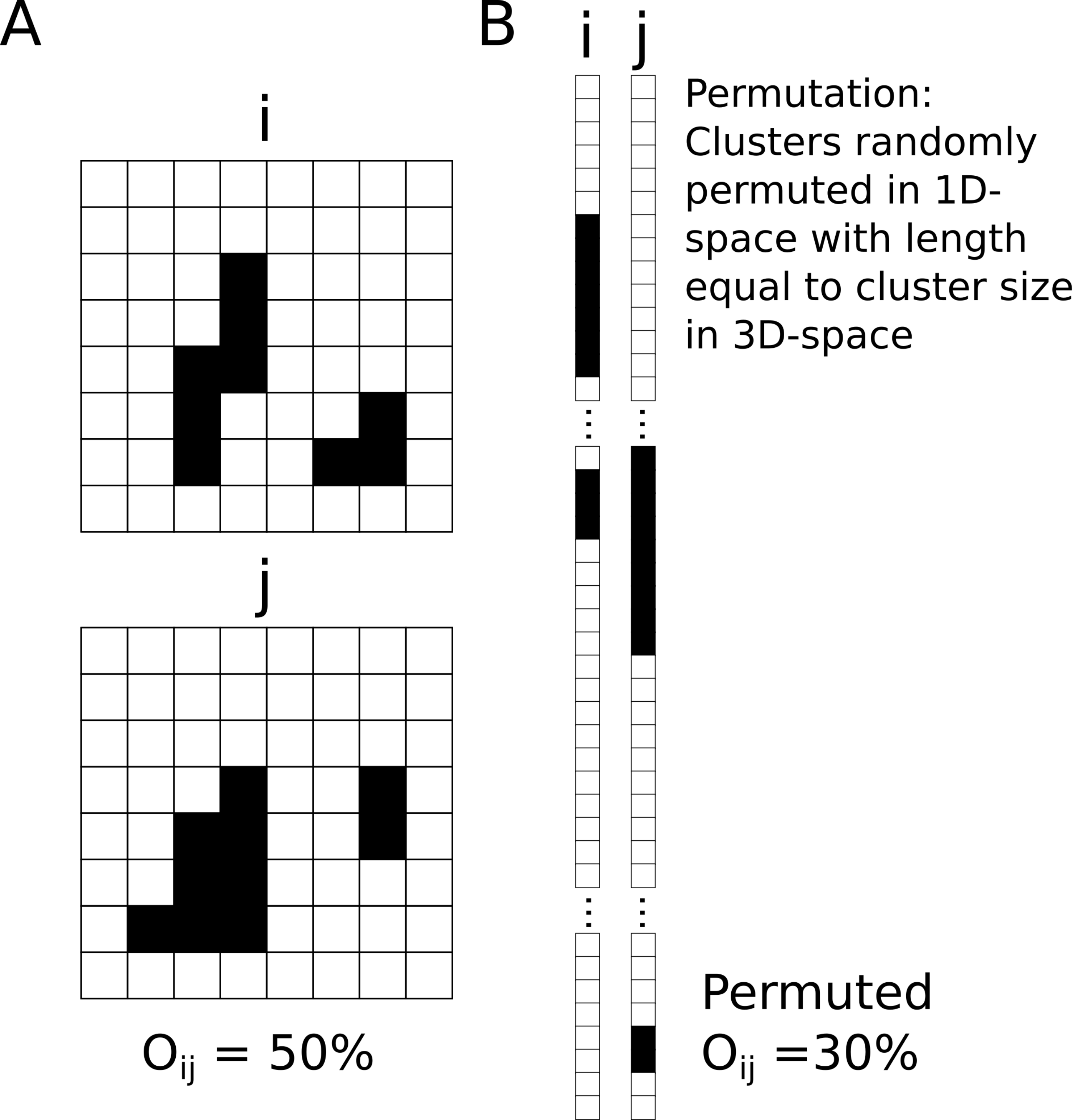
A
B
i
j
Permutation: Clusters randomly permuted in 1D-space with length equal to cluster size in 3D-space
Oij = 50%
Permuted Oij =30%

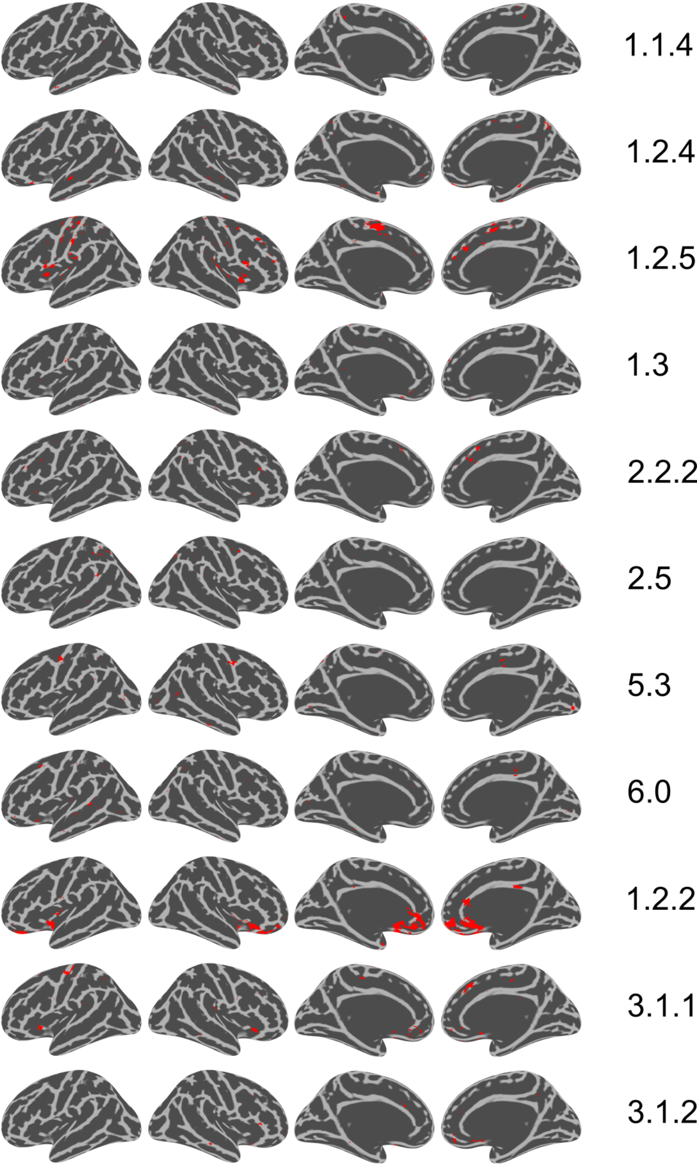
1.1.4
1.2.4
1.2.5
1.3
2.2.2
2.5
5.3
6.0
1.2.2
3.1.1
3.1.2

Name	Network Type	InfoMap Cluster Name	Clustering coefficient	Naming weight
access	PCN	C4.5	0.0140764074	0.5554267489
action	PCN	C5.2	0.0694300656	0.6573649761
action observation	PCN	C5.2	0.0475485823	0.7851630328
adaptive	PCN	C8	0	0.1
affect	PCN	C1.2.3	0.0783486652	0.3730699566
analyzing	PCN	C9	0	0.1
arousal	PCN	C1.2.3	0.0899119169	0.4858089315
assessment	PCN	C1.3.2	0	0.5963026727
associative	PCN	C10	0	0.1
attention	PCN	C3.3	0.0354702979	0.4515667293
attentional control	PCN	C2.2.2	0.0091553283	0.4648836466
auditory	BCN	C4.2	0	0.6991811114
autobiographical memory	PCN	C1.1.3	0.0678352664	0.7728812983
awareness	PCN	C1.3.2	0	0.5845668457
basal ganglia	BCN	C1.2.5	0	0.6895558226
belief	PCN	C1.1.3	0.0432183556	0.5869316549
body	PCN	C2.4	0.087532875	0.156440187
categorization	PCN	C4.5	0	0.6556935009
cerebellar	BCN	C5.1	0	0.7881427223
choice	PCN	C3.1.2	0.0149457054	0.3082115069
coding	PCN	C4.5	0	0.6725713029
cognitive control	BCN/PCN	C3.3	0.0524483481	0.3566528257
competing	PCN	C2.2.2	0.0130307435	0.4565242456
concept	PCN	C11	0	0.1
confidence	PCN	C1.2.4	0.023358568	0.3237275665
conflict	PCN	C3.3	0.0116713996	0.3079735143
context	PCN	C1.1.2	0	0.5671520243
control	PCN	C12	0	0.1
coordination	PCN	C5.2	0.0879594708	0.5983483796
decision making	PCN	C3.1.2	0.0283521394	0.4946301383
default mode	BCN	C1.1.3	0.0819510817	1.1
detection	PCN	C1.2.4	0	0.4851282748
discrimination	PCN	C5.2	0.0076902366	0.3728089557
distress	PCN	C1.2.4	0	0.6314552432
domain general	BCN/PCN	C3.4	0.0216040411	0.5166558556
dorsal attention	BCN	C1.1.2	0.0281701579	0.5957855591
effect	PCN	C13	0	0.1
emotion	PCN	C1.2.3	0.1683662644	0.8721270822
emotion regulation	PCN	C1.2.3	0.0569086349	0.5072212151
empathy	PCN	C1.1.3	0	0.5817097379
endogenous	PCN	C5.3	0	0.5564843658
engagement	PCN	C6.2	0	0.6496217967
episodic memory	PCN	C1.2.1	0.0598103765	0.8054931805
evaluation	PCN	C1.1.3	0.0326566736	0.1
examination	PCN	C1.2.4	0	0.5966892355
executive control	BCN/PCN	C3.3	0.0369276782	0.4670265389
expected	PCN	C3.1.2	0.0621555137	0.3268610846
eye movement	PCN	C5.3	0	0.5720603066
face	PCN	C2.4	0.0534529718	0.7508713284
fear	PCN	C1.2.3	0.1007831448	0.6470615087
feedback	PCN	C3.1.2	0.0574716842	0.6765109476
fronto parietal	BCN	C3.3	0.0413000123	0.835020129

fronto temporal	BCN	C4.5	0.078798611	0.568304723
frontostriatal	BCN	C1.2.5	0	0.5406236228
goal	PCN	C3.4	0	0.4415465429
hippocampal	BCN	C1.2.1	0.0983503985	0.6411473045
idea	PCN	C6.1	0	0.1
identification	PCN	C1.1.2	0	0.5928484619
imagery	PCN	C5.2	0.0739815459	0.5249303174
imitation	PCN	C5.2	0.0345645823	0.4623306291
inference	PCN	C1.1.4	0.0203727293	0.5576005099
inhibitory	PCN	C1.1.2	0	0.6012090516
initiation	PCN	C6.1	0	0.5819885541
integration	PCN	C4.5	0.0237505055	0.1
intelligence	PCN	C5.3	0	0.5445212823
interacting	PCN	C1.3.2	0.0045717657	0.6154977755
interoceptive	PCN	C7.2	0	0.1
interpretation	PCN	C6.2	0	0.5490491031
knowledge	PCN	C1.1.3	0.021485018	0.4803724434
language	PCN	C4.3	0.1834464497	1.1
language comprehension	PCN	C4.3	0.1307531615	0.6828920235
learning	PCN	C3.1.1	0.0435928698	0.4261828387
lexical	PCN	C4.3	0.1187070865	0.419603448
limbic	BCN	C1.2.3	0.1612421855	0.8340639698
maintenance	PCN	C3.3	0.0265384611	0.4566545547
meaningful	PCN	C2.5	0	0.5205987087
memory	PCN	C1.2.1	0.0351675827	0.7921845949
memory encoding	PCN	C1.2.1	0.0302830619	0.3572790317
memory retrieval	PCN	C1.2.1	0.0386624728	0.5886877746
mentalizing	PCN	C1.1.3	0.0737236545	1.0011405992
mind	PCN	C1.1.3	0.0583278629	0.7586208446
mirror neuron	PCN	C5.2	0.0562506587	0.2881358197
monitoring	PCN	C3.2	0.0133394637	0.3647097516
morphological	PCN	C1.1.4	0	0.5849975481
motion	PCN	C2.4	0.0570101063	0.6580795586
motivational	PCN	C3.1.1	0.0396188262	1.1
motor	BCN	C5.2	0.1331292114	0.872511867
motor control	PCN	C5.2	0.0450101534	0.2005886826
motor imagery	PCN	C5.2	0.0798489088	1.1
naming	PCN	C4.3	0.0362161438	0.3919390399
navigation	PCN	C1.2.1	0.0749346386	0.6367526512
negative affect	PCN	C1.2.3	0.0560612839	0.6123396739
nociceptive	PCN	C7.1	0	1.0276374045
object recognition	PCN	C2.4	0.0326792134	0.1513537469
observation	PCN	C2.3	0.0552247645	1.0528860032
occipitotemporal	BCN	C2.4	0.035156823	0.3167856574
olfactory	PCN	C1.2.2	0.0903749289	0.4526758683
orientation	PCN	C5.3	0.0040430864	0.4361312253
orienting	PCN	C2.3	0.0120490933	0.4737280711
pain	PCN	C7.1	0	1.1
parietal frontal	BCN	C2.5	0.0193159499	0.5175268501
perspective	PCN	C1.1.3	0	0.5758549128
phonological	PCN	C4.3	0.1107754485	1.0478951067
planning	PCN	C2.3	0.0499505915	0.5562887969
practice	PCN	C1.3.3	0.0161648762	0.586075539

prediction	PCN	C3.1.1	0.0432421311	0.1
prefrontal parietal	BCN	C2.2.1	0.0361746515	0.4856188344
prefrontal temporal	BCN	C1.2.4	0	0.599779473
preparation	PCN	C2.3	0.0812177002	0.6784203271
reading	PCN	C4.1	0.0982142374	0.6871296609
reasoning	PCN	C2.2.1	0.0214747359	0.4399807148
recognition	PCN	C2.4	0.0558794695	0.6231056953
recognition memory	PCN	C1.2.1	0.0452700096	0.3753963098
recollection	PCN	C1.2.1	0.0515757305	0.6672996109
relevance	PCN	C14	0	0.1
representation	PCN	C2.3	0.0225482526	0.1
response inhibition	PCN	C3.2	0.0357734995	0.5131769168
retrieval	PCN	C2.2.1	0.0436023916	0.7316949484
reward	PCN	C1.2.2	0.0159544756	0.7113106953
rhythm	PCN	C5.1	0.0168009072	0.1
risk	PCN	C3.1.1	0.0379713096	0.3679888587
salience	BCN/PCN	C1.1.2	0.016155883	0.3251996586
self	PCN	C1.1.1	0.0904836132	0.5299466891
self referential	PCN	C1.1.1	0.0503098692	0.6245867668
semantic	PCN	C4.1	0.305047213	0.943074146
semantic memory	PCN	C1.2.1	0.0665824105	0.5657429865
sensorimotor	BCN	C2.1	0.0952897207	0.6476555351
sexual	PCN	C1.3.1	0.0252476604	0.5392644562
sleep	PCN	C6.1	0	1.1
social	PCN	C1.1.1	0.0540639319	0.6936428932
social cognition	PCN	C1.1.1	0.0578669635	0.4648260207
somatosensory	BCN	C5.1	0	0.6999675226
spatial attention	PCN	C2.3	0.0305221221	0.4706881641
speech	PCN	C4.2	0.0650734721	0.8197763444
speech production	PCN	C4.2	0.0867646295	0.9098142326
stress	PCN	C1.3.1	0.0032956995	0.5511159502
subcortical	BCN	C1.2.5	0	0.5234974673
sustained attention	PCN	C1.3.1	0	0.5976105026
switching	PCN	C2.2.1	0.0180404207	0.2952019844
task control	PCN	C4.6	0	0.6918590308
task positive	BCN/PCN	C1.1.2	0	0.595783531
temporal frontal	BCN	C4.4	0.1029045323	0.5641856952
temporal parietal	BCN	C1.1.1	0.071362689	0.6171794319
visual	BCN	C2.1	0.1422259589	1.1
visual attention	PCN	C2.1	0.0391201277	0.2711044162
visuomotor	BCN	C2.1	0.0478979695	0.4349724301
visuospatial	PCN	C2.1	0.0302909308	0.483441113
working memory	PCN	C2.2.1	0.0283332937	0.9395581955

Search terms
access
action
action observation
adaptive
affect,affective
analyzing
arousal
assessment
associative,association,associations
attention,attentional
attentional control
auditory
autobiographical memory
awareness
basal ganglia
belief
body
categorization
cerebellar,cerebellum
choice
coding
cognitive control
competing,competition
concept
confidence
conflict
context
control,controlling
coordination
decision making,decision
default mode,default
detection
discrimination
distress
domain general
dorsal attention
effect
emotion regulation
emotion,emotional
empathy
endogenous
engagement
episodic memory
evaluation
examination
executive control,executive function,executive
expected,expectancy
eye movement
face
fear
feedback
fronto parietal,frontoparietal,frontal parietal

fronto striatal,fronto striatal
fronto temporal,frontotemporal
goal
hippocampal
idea
identification
imagery
imitation
inference
inhibitory,inhibition,inhibitory control
initiation
integration
intelligence
interacting
interoceptive
interpretation
knowledge
language comprehension,comprehension
language,linguistic
learning,learned
lexical
limbic
maintenance
meaningful
memory
memory encoding
memory retrieval
mentalizing
mind
mirror neuron,mirror
monitoring,monitor
morphological
motion
motivational
motor control
motor imagery
motor,movement
naming
navigation
negative affect
nociceptive
object recognition
observation,observing
occipitotemporal
olfactory
orientation
orienting
pain
parietal frontal
perspective
phonological
planning
practice

prediction,predictions
prefrontal parietal
prefrontal temporal
preparation,preparatory
reading
reasoning
recognition
recognition memory
recollection
relevance,relevant
representation
response inhibition
retrieval
reward
rhythm
risk
salience
self
self referential
semantic
semantic memory
sensorimotor,sensory motor
sexual,sex
sleep
social
social cognition,social cognitive
somatosensory
spatial attention
speech
speech production
stress
subcortical
sustained attention
switching,switch
task control
task positive,task relevant
temporal frontal
temporo parietal,temporo parietal,temporoparietal,temporal parietal
visual attention
visual,visual perception
visuomotor
visuospatial
working memory
